# Comparison of measurements made on dry bone and digital measurements in Anatomage for the sacral bone in a Spanish population

**DOI:** 10.1038/s41598-023-48013-8

**Published:** 2023-11-23

**Authors:** B. Gaya-Sancho, D. Sanjuan-Sánchez, A. Ráfales-Perucha, L. Zaurín-Paniagua, B. Sáez-Gutiérrez, S. Galarreta-Aperte

**Affiliations:** 1 Health Science Faculty, Universidad San Jorge, Zaragoza, Spain; 2 Campus Universitario Villanueva de Gállego, Autov. A-23 Zaragoza - Huesca, Km. 299, Villanueva de Gállego, 50830 Zaragoza, Spain

**Keywords:** Anthropology, Anatomy

## Abstract

The use of osteometry for human identification is a key element in the field of forensic sciences. Currently, the osteometry focuses on the use of digital techniques such as photography or 3D scans, to study and measure bones, offering advantages like easy access, preservation of bones, and worldwide collaboration possibilities. The study aims to analyze whether digital tools such as Anatomage can be used to collect reliable data. The study compares measurements of the sacral bone from 41 individuals from Orgiva Collection using both traditional and digital methods. The variables analyzed were described previously, including landmarks and positions, and were coded by differentiating the measurements between dry bone (caliper) and digital measurement (Anatomage). Results indicate minimal differences between digital and dry bone measurements, with only one variable showing a significant differences in the effect size analysis (d > 0.80). The TEM analysis showed four variables as non-acceptable (rTEM > 1.5), possibly due to the landmark location or the experience using the tool to locate landmarks. Digital resources are valuable for morphometric evaluations and human identification within forensic sciences. However, caution is necessary to ensure accurate landmark localization and validate these tools across various bone types and larger sample sizes.

## Introduction

Osteometry uses measurements of the human body for different purposes, such as human identification, by attributing certain characteristics to an individual. Thus, it is a very useful tool in the field of forensic sciences^1–3^. Forensic anthropology uses measurements of bone to provide details such as sex, stature, and/or age; this information offers a greater probability of making a positive identification of the individual^4–6^. Several bone structures, including both the axial and appendicular skeletons, have been used to estimate sex, stature, or population affinity^7^. Bones such as the skull, pelvis, and teeth can be measured; each of them has a certain percentage of reliability for correct classification^1,8–10^.

In general, bones are measured by using validated instruments such as a caliper (King’s Foot) or an osteometric board. Researchers have used these tools to measure dry bones and have established *landmarks* with the specific purpose of human identification^11–13^. However, there are some problems with using dry bone: the conservation of the bones may not be adequate, and measurement of dry bone could lead to deterioration, wear, or alteration of its morphology (taphonomic aspects). Therefore, measuring dry bone can result in measurement bias in the defined variables^14^.

Currently, digital tools are implemented in forensic anthropology for identification purposes. Researchers began to use digital techniques for identification, including superposition of images, three-dimensional (3D) computer reconstructions of individualizing characteristics, and digital reconstruction of facial morphology^15–17^. In addition, this field of study uses diagnostic imaging modalities to obtain even more information than what could be obtained by measuring dry bone. Moreover, 3D scanners can replicate the bone in a digital format; these images allow analyzing the structure of bones with different software programs to facilitate the identification of individuals and develop or test a method^18–20^.

There are several software programs that allow analyzing diagnostic images, photographs, or 3D scans of bones for forensic purposes or for the analysis of different pathologies^21,22^. The use of diagnostic images has the advantage of faithfully representing the structure, although the measurements may have a margin of error depending on the quality of the processing. On the other hand, they have the advantage of being stored in centralized databases. This encourages multidisciplinary and international work^23–25^.

Likewise, these digital techniques help to preserve recovered bones because measurements can be made by using the digital images rather than the actual bone. In addition, images can be accessed anywhere in the world and thus analysis would not be dependent on the location of the bone^24^. However, the tools must be validated, and protocols must be standardized before they could be applied to forensic anthropology^24,26^. Among these tools, the Anatomage dissection table stands out as a teaching tool that has research potential thanks to all the tools it offers: from diagnostic image study to data acquisition with measurements. This type of tool is growing and expanding, so all its functionalities need to be tested^27^.

The study aims to analyze whether digital tools such as Anatomage can be used to collect reliable data. Thus, we analyzed the effectiveness of using digital tools for bone osteometry compared with traditional measurements on dry bone.

## Methods

We carried out a comparative study of different methods to measure bones. We evaluated the sacral bone because we have a database of measurements of this structure for a population from Órgiva (Granada, Spain)^28^ at our disposal. Furthermore, we scanned the same structures with an Artec 3D scanner to generate digital images that could be used for measurements. The database consists of 60 individuals for which we have the age, sex, and a record of all measurements on the sacral bone. All the methods were carried out in accordance with relevant guidelines and regulations considering that the sample were donated by Órgiva Cemetery and consisted of skeletal remains from a mass grave. The mass grave was exhumed, and the skeletal remains were donated to the University of Granada. The eligibility of the final sample had to meet the following selection criteria: to have all the variables to be analyzed for each of the structures.

An Excel database containing the main variables identified by Gaya-Sancho^29^ was used for comparison with the digital measurements. The original measurements were made with a sliding caliper and only those with a measurement agreement greater than 0.9 (ICC classification) were included (Gaya-Sancho et al.^29^). These measurements were defined by the mentioned author anatomically in order to locate the landmarks manually and visually.

We used the Anatomage (Anatomical Dissection Table) to make the same measurements (under the same definitions for landmarks) of digital images of the sacral bones as had been done on the actual bone. The measurements were performed on three-dimensional structures scanned directly on the Anatomage® dissection table, and the defined landmarks could be located by rotating and moving the structure to ensure correct accuracy for location.

We registered the variables with a number (1 or 2) to identify whether it is the dry bone measurement (1) or the digital measurement (2). Table 1 shows the definition and acronyms of each variable as defined by Gaya-Sancho et al.^29^. Each variable includes lateralization (L: Left or R: Right) and all the variables were taken in mm.

**Table 1:** Definition for each variable including abbreviation and name.

SCW	Maximum sacral canal width	Maximum width established between both attachment points of the body and the transverse processes in S1 within the canal
TL	Maximum transverse line width	Maximum crest distance between the sacral vertebrae. Includes superior (S), Mid-superior (MS), Mid-inferior (MI), and Inferior (I)
LC	Maximum lateral crest length	Maximum length from the upper end of the transverse process from S1 to the lower end of S5
MSW	Maximum sacral width	Distance between the most lateral points of the wings
ML	Maximum length	Length from the upper midpoint of the promontory to the apex of the sacrum, taken in the sagittal plane
AFH	Height of the articular facet	Distance from the highest point to the lowest of the articular facet of the articular process
ASH	Height of the auricular surface	Distance between the upper and lower points of the articular facet, taken in anatomical position

We recorded the measurements in situ by using the same database that had been configured in JAMOVI v.2.3.18. After we recorded and compiled the variables, we analyzed the normality of the data with the Shapiro–Wilk test. Because the data had a normal distribution, we used Student's t test for paired samples to compare the measure of each variable taken from dry bone and taken from digital images. We considered p < 0.05 to be statistically significant. Finally, we determined the effect size by calculating Cohen's d. This approach allowed us to analyze the power of any significant differences^30,31^.

Finally, a study of the TEM and the correlation coefficient between the different observations was carried out using RStudio to evaluate the differences in measurement and observation between the different methods^32,33^ following the International Society for the Advancement of Kinanthropometry (ISAK) recommendations. The cut-off point for interpretation is set at 1.5% for RTEM to be considered acceptable for a beginner anthropometrist^34^.

## Results

We recorded measurements of the variables for 41 of the 60 available individuals. We excluded the remaining 19 individuals because they did not have a record of all the measures for each structure. Landmark localization was performed without incident according to the definition of Gaya-Sancho et al.^29^. The ability to move the structure to locate these landmarks facilitated the data collection process.

Table 2 shows the descriptive statistics (mean and standard deviation) for each variable. For all the variables obtained with the digital tool Anatomage, the mean tends to be higher when considering the scans. On the other hand, the standard deviation values of each variable, obtained both manually with the caliper and digitally, remain more homogeneous without showing any trend.

**Table 2:** Descriptive statistics of the bone structures.

	N	Mean	Median	SD
SCW1	41	29.9	29.8	2.93
SCW2	41	30.1	29.6	3.07
STL1	41	33.0	32.9	4.08
STL2	41	32.8	32.8	4.00
MSTL1	41	29.7	30.5	2.80
MSTL2	41	29.6	30.2	2.80
MITL1	41	27.7	27.4	2.75
MITL2	41	27.6	27.2	2.79
ITL1	41	26.9	26.5	2.47
ITL2	41	26.8	26.5	2.38
LLC1	41	108.6	108.6	7.02
LLC2	41	108.4	108.6	7.18
RLC1	41	108.8	106.8	7.65
RLC2	41	108.4	106.9	7.60
MSW1	41	115.6	117.5	6.71
MSW2	41	115.0	116.1	6.82
ML1	41	102.0	101.6	10.01
ML2	41	101.8	101.9	10.02
LAFH1	41	16.3	16.2	2.02
LAFH2	41	16.3	16.3	1.63
LASH1	41	59.4	58.8	5.19
LASH2	41	59.3	59.0	5.11
RASH1	41	59.8	59.4	5.60
RASH2	41	59.7	59.5	5.53

*N* number of individuals, *SD* standard deviation.

Table 3 shows the statistical analysis comparing the measurements with dry bone and digital images.

**Table 3:** Statistical analysis of dry bone measurements versus digital measurements.

		T value	P	Mean difference	EE of difference	Cohen’s d
SCW1	SCW2	− 3.08753	**0.004**	− 0.2239	0.0725	− 0.482
STL1	STL2	5.28430	** < 0.001**	0.2444	0.0462	**0.825**
MSTL1	MSTL2	2.51528	**0.016**	0.0995	0.0396	0.393
MITL1	MITL2	2.91073	**0.006**	0.1376	0.0473	0.455
ITL1	ITL2	1.94983	0.058	0.0912	0.0468	0.305
LLC1	LLC2	1.16470	0.251	0.1749	0.1501	0.182
RLC1	RLC2	1.33923	0.188	**0.3656**	0.2730	0.209
MSW1	MSW2	2.93361	**0.006**	**0.6232**	0.2124	0.458
ML1	ML2	1.31703	0.195	0.2100	0.1594	0.206
LAFH1	LAFH2	0.00420	0.997	7.32e−4	0.1743	6.56E−4
LASH1	LASH2	0.70046	0.488	0.0454	0.0648	0.109
RASH1	RASH2	1.63178	0.111	0.1083	0.0664	0.255

*P* p-value.

The measurements were compared with Student’s t test. Significant differences (p < 0.05) are presented in bold. Cohen’s d represents the effect size.

Of the 12 variables, only five showed significant differences between the dry bone and digital image measurements. However, only one of these variables had a large effect size (STL); the other four variables had a low (SCW) or moderate (MSTL, MITL, and MSW) effect size. The effect size analysis showed that, despite finding significant differences, the impact of these differences is not high except for STL. This large effect size implies larger differences between the STL means, which could be due to the location of the landmarks themselves, the experience of using the digital tools to locate and thus measure the landmarks, or the variability that could be found in digitizing bones.

Additionally, Table 3 shows the mean differences, which provide some interesting insights. First, although MSTL was significantly different between the dry bone and digital image measurements, the mean difference was only 0.099 mm. Second, the mean difference for most variables was < 0.25 mm (except for RLC and MSW), even when there was a significant difference between the measurement methods.

Table 4 shows the result for the TEM analysis and the reliability between observations. The “Non acceptable” of this error analysis based on rTEM is observed for four of the variables. Moreover, these variables coincide with those that show significant differences between observations (Table 3). This could be explained by the experience using the tool for this purpose.

**Table 4:** Statistical analysis of technical error measurements and coefficient correlation (r).

		TEM	rTEM (%)	Classification	r
SCW1	SCW2	1.013	3.4	Non acceptable	0.989
STL1	STL2	1.106	3.4	Non acceptable	0.998
MSTL1	MSTL2	0.450	1.5	Acceptable	0.996
MITL1	MITL2	0.622	2.2	Non acceptable	0.994
ITL1	ITL2	0.413	1.5	Acceptable	0.993
LLC1	LLC2	0.413	0.6	Acceptable	0.991
RLC1	RLC2	1.655	1.5	Acceptable	0.974
MSW1	MSW2	2.821	2.4	Non acceptable	0.980
ML1	ML2	0.950	0.9	Acceptable	0.995
LAFH1	LAFH2	0.003	0.0	Acceptable	0.834
LASH1	LASH2	0.205	0.3	Acceptable	0.997
RASH1	RASH2	0.490	0.8	Acceptable	0.997

Significant values are in underlined.

*TEM* technical error measurement, *r* correlation coefficient.

The variable MSTL shows significant differences, although the TEM analysis provides an acceptable result for these observations. In this case, the variable shows a small deviation in the mean difference (Table 3) between the two methods of measurement. According to the formulas proposed for the sacrum^28,29^, this variable is not included in those that give a higher percentage of reliability, so its applicability is not compromised.

For all these reasons, digital tools make it possible to analyze structures despite the differences observed in most of the variables analyzed. These digital tools make it easier to obtain data by allowing resources to be manipulated, zoomed, and managed efficiently.

## Discussion

The use of digital measurements within forensic sciences is growing. Hence, the effectiveness of the available software programs and tools must be evaluated to ensure their accuracy, as traditional measurements have proved to be. Validating these digital tools could facilitate the creation of new methods of human identification.

Diagnostic images and 3D models of bones have begun to be used because they faithfully represent the scanned structure and allow a macroscopic and morphometric analysis of them. Abbeg et al.^35^ analyzed skulls using computed tomography (CT) and a 3D scanner; they highlighted that there may be discrepancies between the measurements, similarly to what we have found in the present study. Thus, it is necessary to reconsider, on some occasions, the landmarks that are used. Landmark localization as determined by Lou et al.^36^ and Jerkovic et al.^37^, may be inaccurate at first, although landmark localization improves with repeated practice. The differences found in our results could be caused by this phenomenon. Therefore, further research with a larger number of structures is recommended.

Sevillano et al.^38^ demonstrated that there is a high correlation between traditional and virtual measurements for the models they analyzed. Colman et al.^39^, Banik et al.^40^, and Citardi et al.^41^ analyzed the differences between dry bone measurements and 3D virtual models. Although they obtained variable results, there was a generally good agreement between the observations taken digitally and those taken on dry bone. This means that the use of new digital tools and virtual 3D techniques can currently provide added value to identification in forensic anthropology.

Researchers have also used digital tools, images of diagnostic tests, and resources such as artificial intelligence (AI) to evaluate and/or estimate identifying aspects of humans, such as sex and age. This research suggests that the implementation of neural networks can enhance and greatly benefit the work of anthropologists^42,43^. Some researchers have used 3D models, diagnostic tests, or 3D structures generated by scanners for validation or to create models to estimate individual characteristics; these authors reported results ranging from 74 to 91.40% success for the discriminant functions developed to estimate sex^44,45^.

Based on our results and the literature, we recommend continuing validating digital tools by examining other bones. All these results suggest that the integration of digital tools for the study of bone remains can be equated to that carried out with in dry bone. In addition, the availability of these digital files of digitized bones would help to perform the analysis even when the bone remains are not in the same location as the researcher, since they can be sent and managed through digital files.

These efforts could validate the virtual dissection table as a potentially useful tool for handling, studying, and measuring bones in forensic anthropology. These efforts could help to create virtual datasets of bone collections so that these structures can be studied in the future as they can be digitally preserved^46^.

## Conclusions

Digital resources are useful to analyze bone because they allow measuring structures for morphometric evaluation and human identification within forensic sciences. However, these tools must be used with caution because the 3D manipulation of these structures, under a screen, could skew the information and the exact location of the landmarks in the structures. We only found a few significant differences between the dry bone and digital measurements, and they had a relatively low impact level. Nevertheless, digital tools must continue to be validated in larger samples and for different bones, and even anthropometric measurements.

## Data Availability

The data that support the findings of this study are available from the corresponding author, [G-S B], upon reasonable request.
